# A Newly Diagnosed Patient With Human Immunodeficiency Virus (HIV)-Associated Epstein-Barr Virus (EBV)-Positive Diffuse Large B-cell Lymphoma and Esophageal Candidiasis: A Case Report

**DOI:** 10.7759/cureus.59106

**Published:** 2024-04-26

**Authors:** Mina Daniel, Jigar Patel, Maria Kamel, Darshan Roy

**Affiliations:** 1 Internal Medicine, Memorial Hermann Health System, Houston, USA; 2 Gastroenterology, Memorial Hermann Health System, Houston, USA; 3 Medical School, Columbus Central University School of Medicine, Belize City, BLZ; 4 Pathology, Memorial Hermann Health System, Houston, USA

**Keywords:** epstein-barr virus-positive diffuse large b-cell lymphoma, aids-defining illness, gastric mass, gastrointestinal lymphomas, hiv lymphoma, esophageal candidiasis

## Abstract

The link between the Epstein-Barr virus (EBV) and the development of certain types of lymphomas in patients with human immunodeficiency virus (HIV) is of noteworthy clinical importance. Their immunocompromised state increases the risk of cancers such as lymphomas. Gastrointestinal (GI) lymphomas, though, can occur due to the immunosuppression caused by HIV, with diffuse large B-cell lymphoma (DLBCL) being common in this group. This case report describes a case of a patient with a newly diagnosed HIV who initially presented with symptoms associated with EBV-associated DLBCL and with esophageal candidiasis. This case report highlights the need for increased awareness of HIV-related complications and the importance of close follow-up. In addition, despite advancements in highly active antiretroviral therapy (HAART), acquired immunodeficiency syndrome (AIDS)-related lymphomas continue to be a concern requiring treatment approaches.

## Introduction

Epstein-Barr virus (EBV) is the cause of different types of lymphomas, such as Burkitt's lymphoma and Hodgkin lymphomas, in patients with human immunodeficiency virus (HIV). The incidence of cancers like lymphomas significantly rises in the immunocompromised HIV patients. Although gastrointestinal (GI) lymphomas are uncommon, these patients are more prone to non-Hodgkin lymphomas (NHL), including diffuse large B-cell lymphoma (DLBCL), the most common type seen in this group. In our case report, we are presenting a case of an HIV patient diagnosed with EBV DLBCL and esophageal candidiasis, highlighting the complexities of complications related to acquired immunodeficiency syndrome (AIDS) [1-4].

The early initiation of highly active antiretroviral therapy (HAART) leads to improvements in managing HIV infection and results in lower viral load and higher CD4 T lymphocytes, which, in turn, decrease infections related to HIV. It is crucial to grasp the connection among HIV infection, EBV, and the onset of lymphomas to ensure identification and efficient treatment. This particular instance highlights the significance of early diagnosis of HIV individuals, stressing the necessity for increased awareness and holistic approaches to tackle AIDS-linked issues like lymphoma development. By clarifying the processes behind HIV-associated lymphomas, healthcare professionals can enhance their ability to identify and handle these illnesses, leading to better results for patients and their quality of life [5-7].

## Case presentation

**Figure 1:**
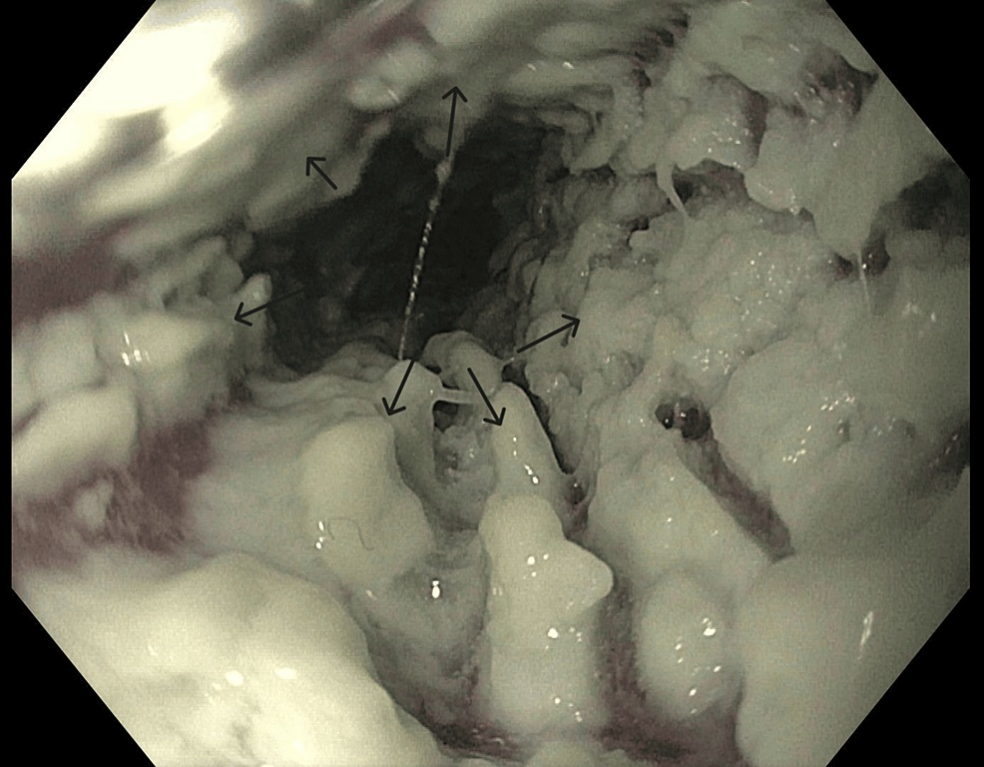
Endoscopic view of the esophageal lumen EGD revealed diffuse white plaques carpeting the esophageal mucosa along the length of the esophageal lumen, compatible with a diagnosis of esophageal candidiasis EGD: esophagogastroduodenoscopy

**Figure 2:**
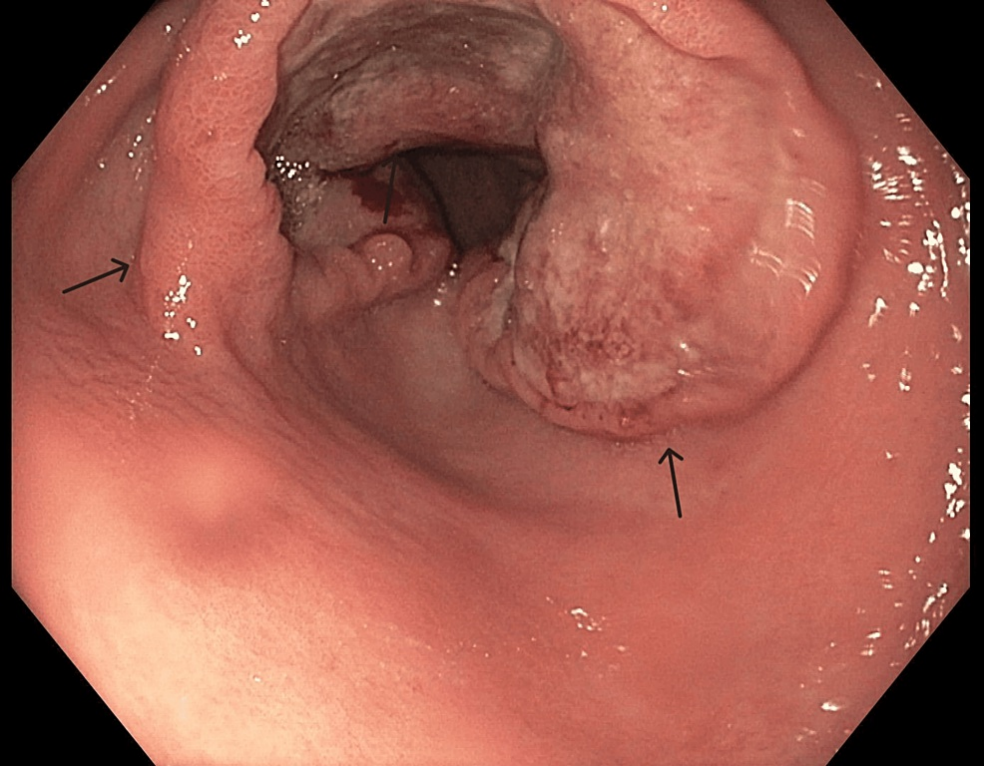
Endoscopic view of ulcerated mass at the gastric antrum EGD revealed a large circumferential ulcerated mass in the gastric antrum extending to the first part of the duodenum EGD: esophagogastroduodenoscopy

**Figure 3:**
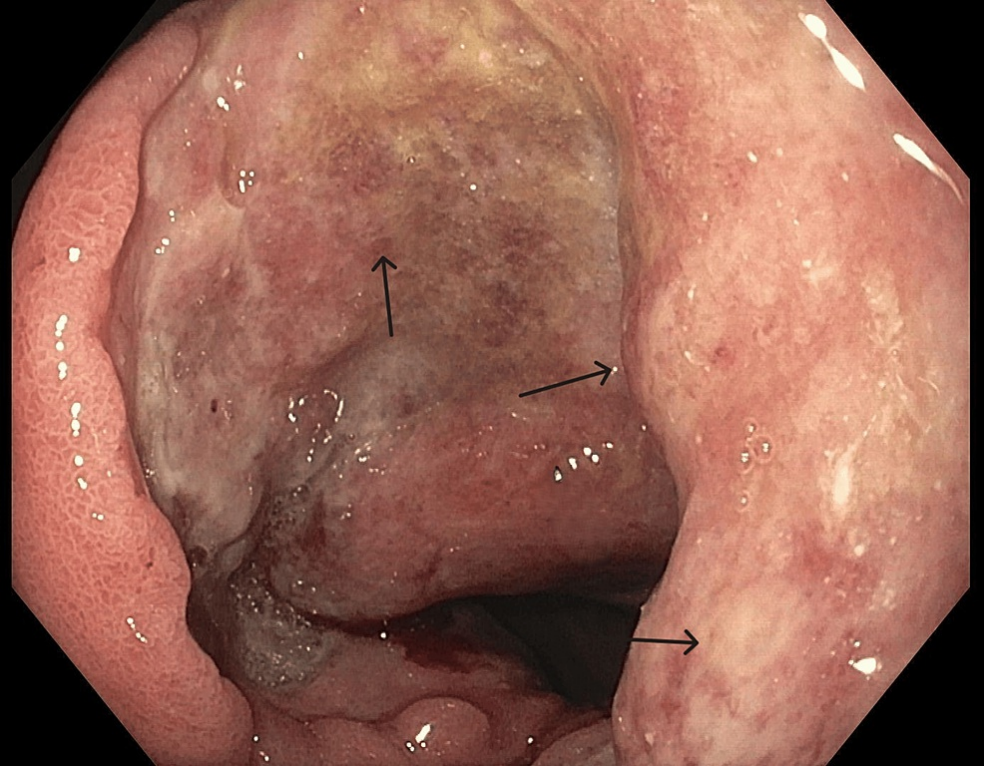
Endoscopic view of ulcerated mass at the gastric antrum EGD revealed a large circumferential ulcerated mass in the gastric antrum extending to the first part of the duodenum EGD: esophagogastroduodenoscopy

**Figure 4:**
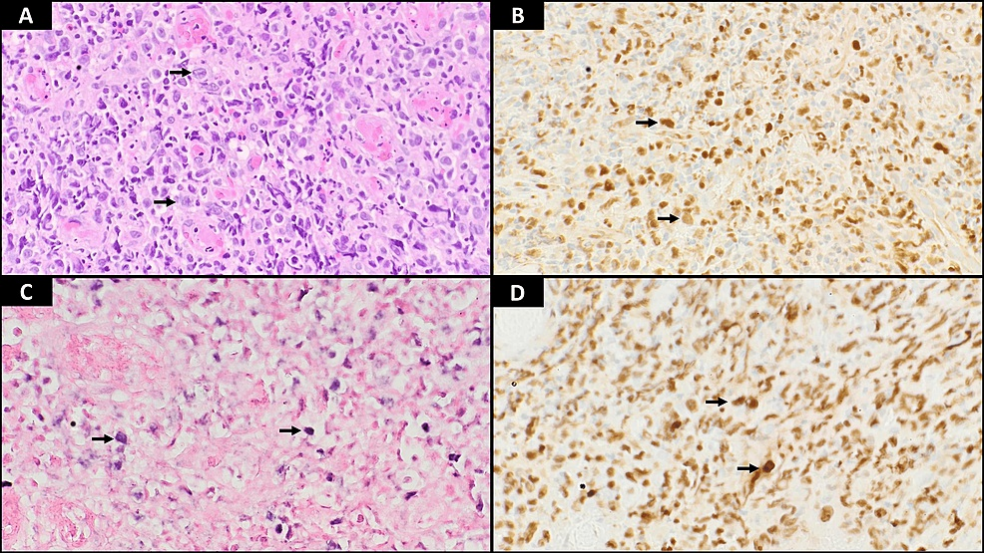
Histopathology analysis from biopsies of gastric mass Gastric biopsy (all images at 400×). (A) H&E-stained section of the gastric mucosa showing large atypical lymphoid cells. (B) Tumor cells show nuclear positivity for pan-B-cell marker PAX5. (C) EBV stain (EBER by in situ hybridization) is positive within the atypical cells. (D) Ki-67 staining shows an increased proliferative rate of 95% H&E: hematoxylin and eosin; EBV: Epstein-Barr virus; EBER: Epstein-Barr encoding region

A 41-year-old male with no significant past medical history presented to the emergency room with intractable symptoms of nausea with vomiting, decreased appetite, and significant weight loss of around 40-50 pounds over the past 1-2 months. He also reported dysphagia and odynophagia symptoms. Previously, he was seen in the emergency room several times within the past few weeks but could not follow up with a gastroenterologist. A CT scan conducted at an outside emergency department revealed irregular circumferential thickening of the gastric antrum, mild diffuse enteritis, and prominent periaortic mesenteric lymph nodes. The patient denied smoking cigarettes, illicit drug use, nonsteroidal anti-inflammatory drug (NSAID) use, and a family history of GI malignancy. Laboratory results revealed an HIV viral load of 350,000 copies/mL, CD4 T-cell count <20 cells/mm^3^, hemoglobin 13.3 g/dl, hematocrit 38.5%, and platelets 342,000/ul, and a comprehensive metabolic panel was within normal limits except for a slightly decreased albumin level of 3.2 g/dl. Esophagogastroduodenoscopy (EGD) revealed severe esophageal candidiasis (Figure 1) and a large circumferential ulcerated mass in the gastric antrum extending to the duodenum (Figure 2 and Figure 3). Random gastric biopsies were negative for *Helicobacter pylori* infection. Pathology results from the gastric mass biopsy indicated EBV-positive DLBCL (Figure 4), confirmed by fluorescence in situ hybridization (FISH) studies, with positive immunostaining for *Cytomegalovirus* and concurrent detachment of *Candida* species. At this point, HIV testing was pursued, which was positive. The patient was initiated on fluconazole, and antiretroviral therapy (ART) was planned to address the probable AIDS diagnosis.

Given the case's complexity, a team of experts from infectious diseases, gastroenterology, and oncology came together to manage it. The treatment included a 21-day course of antifungal treatment and arranging for outpatient oncology follow-up to stage and manage the lymphoma. It was important to monitor the patient's progress and address any issues that may arise. This case highlights how AIDS-related conditions like lymphoma and esophageal candidiasis are interconnected, emphasizing the need for a well-coordinated approach. 

## Discussion

GI lymphomas are uncommon, with 0.8-1.2 cases per 100,000 people yearly. They represent a fraction (1-4%) of tumors found in the stomach, small intestine, and colon; the GI tract is affected secondarily in 10% of patients. The stomach is commonly affected, accounting for 68-75% of cases, followed by the small intestine (9%), ileocecal region (7%), and rectum (2%) [1].

In cases involving HIV infection, DLBCL is the prevalent type of lymphoma, making up approximately half of all lymphomas in this group. Among these instances, around 30-40% are associated with the EBV. DLBCL differs from Burkitt's lymphoma as it impacts individuals with compromised systems. The initiation of HAART has reduced the likelihood of developing NHL and enhanced outcomes for HIV individuals with DLBCL [2].

Gastric lymphoma is a form of cancer that impacts the stomach, representing about 3% of all stomach malignancies and 10% of all lymphomas. In individuals with immunosuppression due to HIV, NHL is frequently observed in those with low CD4 count (usually less than 100 cells/mm^3^), high viral load, advanced age, and prior AIDS-related conditions. Gastric lymphomas tend to be more prevalent among men aged between 50 and 60 [3].

DLBCL accounts for half of all lymphomas in individuals with HIV. EBV is a type of herpesvirus that can establish a lasting infection in B cells. In people with HIV, the compromised immune system can lead to the reactivation of EBV, causing the affected B cells to grow and transform, increasing the likelihood of developing lymphoma. HIV primarily targets CD4 T cells, gradually weakening the system's ability to combat infections. As CD4 cell counts decline, the immune system becomes more vulnerable to reactivating infections [4].

The clinical signs of lymphoma linked to the EBV in HIV patients can manifest as lymph nodes, weight loss, night sweats, and fatigue. Our patient showed symptoms. The diagnosis was delayed because they did not follow up with a gastroenterologist for a thorough assessment. During an EGD, it was discovered that the patient had candidiasis and a large ulcerated mass in the gastric antrum. The final diagnosis of EBV-positive DLBCL was confirmed based on histopathology results and positive EBV staining [8,9].

Patients living with HIV/AIDS who have weakened systems, as indicated by low CD4 cell counts, experience a significant decrease in both their humoral and cellular immunity. This makes them more vulnerable to opportunistic infections (OIs) caused by pathogens like viruses, bacteria, fungi, and protozoa. These infections significantly raise mortality rates among individuals with AIDS. CD4 cells fight infections by releasing cytokines that activate cells and help establish long-term immunity. It is important to prioritize infection prevention for those affected by HIV/AIDS [10].

While the occurrence of OIs has reduced with the introduction of HAART, they still pose a risk of hospitalizations and deaths related to HIV for those who have not started or continued HAART. Generally, individuals with competent immune systems do not fall ill from OIs [10].

The arrival of ART in care has revolutionized the progression of HIV-1 infection. It has led to a reduction in HIV-1 levels in the blood, an increase in CD4 T-lymphocyte counts, and a decrease in HIV-1-related infections. Consequently, individuals living with HIV-1 are experiencing a 75% decrease in AIDS-related deaths, with their life expectancy following infection now extending to 30-40 years, signifying an enhancement in quality of life. Despite these advancements, the risk of developing AIDS-related cancers remains higher compared to the population [11].

Before the availability of ART, the incidence of NHL among HIV-1 patients was much higher. The adjusted NHL incidence rate was estimated to have decreased from 6.2% before ART to 3.2% after ART, with a decline in the five-year NHL incidence from 3.8% to 2.2%. This reduction in NHL occurrence contrasts with the drop in Kaposi sarcoma (KS), which is associated with herpesvirus 8. The incidence rate of KS decreased from 14.3% before ART to 1.8% after ART. While ART has significantly changed how HIV-1 infection progresses, NHL remains the cancer-defining AIDS. The decrease in NHL occurrence varies among its subtypes. There is a drop in central nervous system lymphoma compared to the minor decrease in other subtypes [11].

HAART aids in boosting the system by inhibiting replication and raising CD4 cell counts to target the root cause of weakened immunity. Individuals with HIV-related DLBCL receive treatment to those with non-HIV related. The successful approach for DLBCL involves a blend of rituximab, cyclophosphamide, doxorubicin, vincristine, and prednisone (R-CHOP). It is highly advised to combine chemotherapy with immunochemotherapy for better results. In cases where a patient shows resistance to treatment or experiences a relapse, alternative treatment options are considered. This may be followed by stem cell transplantation [5-7].

The management of B-cell lymphomas depends not only on how advanced they are but also on specific disease traits like growth speed (slow or fast) and molecular subtype. While DLBCL can be aggressive, some patients can achieve long-term survival but incomplete cure through chemotherapy. Germinal center B-cell (GCB)-like DLBCL patients usually respond well to R-CHOP given every 21 days. Rituximab targets CD20, a protein in B cells. On the other hand, activated B-cell(ABC)-like DLBCL has less favorable responses to R-CHOP treatment [12].

In general, the outlook for NHL hinges on various factors such as disease stage, histopathology, lymph node and extranodal involvement, patient age, and overall health status. Doctors track treatment progress through evaluations, lab tests, and imaging scans. The International Prognostic Index aids in predicting outcomes by considering factors like age above 60 years old; Eastern Cooperative Oncology Group performance score greater than 2; elevated lactate dehydrogenase (LDH) levels in blood tests; stage III or IV disease at diagnosis; and involvement of sites, outside lymph nodes linked with lower overall survival rates [12].

Up to 50% of individuals with DLBCL can be effectively treated if they go into remission following R-CHOP treatment. Those with the GCB subtype usually have better outcomes than those with the ABC subtype [12].

Efforts from healthcare professionals, including social workers, are essential in preventing OIs in people with HIV/AIDS. Starting HAART early is vital for preventing these infections [10].

Other preventive measures include vaccination and screening for co-infections to reduce exposure risks and educate patients about these risks. Regular screening for infections associated with HIV diagnoses like syphilis, chlamydia, gonorrhea, tuberculosis, and hepatitis A, B, and C is highly recommended for individuals recently diagnosed [10].

Patients with a low CD4 cell count are recommended to undergo treatment for infections like *Mycobacterium tuberculosis* and *Pneumocystis jirovecii*, along with HAART medication. Treatment can be discontinued once the CD4 cell count surpasses 200 cells/microL for a minimum of six months post-initiating HAART [10].

However, there is currently no proof advocating the necessity of treating against organisms such as *Histoplasma capsulatum*, *Mycobacterium avium complex* (MAC), *Bartonella* species, *Cytomegalovirus*, *Cryptosporidium,* and *Candida* species. These pathogens rarely cause infections in individuals with HIV/AIDS. Additionally, there is a risk of drug interactions when introducing antimicrobial medications alongside HAART. Therefore, promptly starting HAART and sufficiently increasing the CD4 cell count are believed to be enough measures to guard against these organisms [10].

The overall effectiveness of vaccines in enhancing the system of those with HIV/AIDS remains uncertain at this time. The compromised immune response seen in patients with low CD4 cell counts due to HIV/AIDS presents challenges in achieving long-lasting protection through vaccination [10].

Despite the hurdles faced, it is highly recommended for individuals in this category to receive vaccinations as they can help reduce the severity and mortality rates associated with diseases through immunization. Generally speaking, inactivated vaccines are preferred over live attenuated vaccines to minimize vaccine-related complications. If there are worries about insufficient immunity response after vaccinations, getting revaccinated is advised once the CD4 cell count exceeds 200 cells/microL [10].

Individuals with immunocompromised systems, such as those with HIV/AIDS, are at higher risk of infections from different factors like environmental exposure or engaging in risky behaviors. Educating patients on ways to avoid risks, such as staying away from animals and safe sex practices, is crucial. Timely HAART initiation also plays a part in reducing the chances of acquiring infections [10].

## Conclusions

This particular instance shows the interaction between HIV infection, the reactivation of the EBV, and the emergence of lymphomas, highlighting the intricate nature of complications related to AIDS. The patient's diagnosis of EBV-associated DLBCL, along with esophageal candidiasis, emphasizes the critical need to be vigilant for HIV-related cancers, especially in those with advanced immune system suppression.

Despite progress in managing HIV, such as the implementation of HAART, concerns about AIDS-related lymphomas persist, requiring attention and comprehensive care strategies. Collaboration among disease specialists, oncologists, and gastroenterologists is crucial for diagnosing, staging, and treating HIV-associated lymphomas.

Effectively treating HIV-related DLBCL involves a blend of therapy and immunochemotherapy tailored to each patient's requirements. Ongoing research endeavors are vital for understanding the underlying mechanisms behind HIV-linked lymphomas and exploring treatment approaches to enhance patient outcomes.

## References

[1] Harada G, Felipe-Silva A, Nogueira da Silva JG (2014). Early stage primary gastric diffuse large B-cell lymphoma in a young HIV-positive patient. Autops Case Rep.

[2] Shindiapina P, Ahmed EH, Mozhenkova A, Abebe T, Baiocchi RA (2020). Immunology of EBV-related lymphoproliferative disease in HIV-positive individuals. Front Oncol.

[3] Heise W (2010). GI-lymphomas in immunosuppressed patients (organ transplantation; HIV). Best Pract Res Clin Gastroenterol.

[4] Powitz F, Bogner JR, Sandor P, Zietz C, Goebel FD, Zoller WG (1997). Gastrointestinal lymphomas in patients with AIDS. Z Gastroenterol.

[5] Linke-Serinsöz E, Fend F, Quintanilla-Martinez L (2017). Human immunodeficiency virus (HIV) and Epstein-Barr virus (EBV) related lymphomas, pathology view point. Semin Diagn Pathol.

[6] Magangane PS, Mohamed Z, Naidoo R (2020). Diffuse large B-cell lymphoma in a high human immunodeficiency virus (HIV) prevalence, low-resource setting. South African Journal of Oncology.

[7] Verdu-Bou M, Tapia G, Hernandez-Rodriguez A, Navarro JT (2021). Clinical and therapeutic implications of Epstein-Barr virus in HIV-related lymphomas. Cancers (Basel).

[8] Huguet M, Navarro JT, Moltó J, Ribera JM, Tapia G (2023). Diffuse large B-cell lymphoma in the HIV setting. Cancers (Basel).

[9] Dolcetti R, Gloghini A, Caruso A, Carbone A (2016). A lymphomagenic role for HIV beyond immune suppression?. Blood.

[10] Sadiq U, Shrestha U, Guzman N (2023). Prevention of opportunistic infections in HIV/AIDS. StatPearls [Internet].

[11] Petrara MR, Freguja R, Gianesin K, Zanchetta M, De Rossi A (2013). Epstein-Barr virus-driven lymphomagenesis in the context of human immunodeficiency virus type 1 infection. Front Microbiol.

[12] Padala SA, Kallam A (2023). Diffuse large B-cell lymphoma. StatPearls [Internet].

